# The World Rabies Day 2020: Collaborate and Vaccinate

**DOI:** 10.29252/ibj.24.5.263

**Published:** 2020-08-03

**Authors:** Alireza Gholami, Ashkan Alamdary

**Affiliations:** Viral vaccines production unit, research and production complex, Pasteur Institute of Iran, Karaj, Iran

**Keywords:** One Health, Prevention and control, Rabies, Vaccination

## Abstract

Rabies constantly kills 59,000 people annually, mostly in Asia and Africa. Rabies, which is responsible for 99% of human rabies cases, is totally preventable by standard vaccinations. In 2015, a global call for action was made by the WHO, OIE, FAO, and GARC to join forces toward the elimination of dog-transmitted human rabies by the year 2030. All the tools and protocols to reach that target are readily available, and the feasibility of dog rabies elimination has been proven. Countries should drive the changes needed to engage into this global movement. Certainly, countries in the EMR require taking more critical steps to reach the rabies elimination target by 2030. The international awareness campaign of the WRD is an excellent occasion to assess challenges and opportunities toward rabies elimination.

**Rabies by numbers**

Each year, 59,000 human deaths occur from inevitably lethal rabies encephalitis in the world, with the dog being considered as the causative agent of up to 99% of rabies transmission to humans. According to the official data, 150 countries all over the world are contaminated with terrestrial rabies viruses. In Africa and Asia, more than 95% of annual deaths occur, 40% of whom being children under 15 years of age^[^^1^^]^. Despite the fact that rabies is underreported, and estimations of rabies burden are uncertain, this viral disease is still a major public health concern, particularly in less privileged areas in Asia and Africa. There is no cure for clinical rabies and standard PEP; using a safe and an efficacious vaccine is the only effective means for saving exposed peoples’ lives. Disease burden depends on various factors such as ecological, economical and sociocultural conditions. Correspondingly, annualhuman deaths in Central Asia and the Middle East have been reported to be 1875 and 229 cases, respectively. In general, Asia contains the largest proportion of the world’s estimated cost, being caused by premature deaths and PEP. The situation is different in the Americas with 10 deaths caused by dogs and 23 by other animals^[^^2^^]^.

**Rabies in the EMR**

In the EMR of the WHO, nearly 600 million people in 22 countries live with the danger of rabies. Among them, only six countries are considered as a low level risk for the transmission of rabies to human^[^^3^^]^. Fox, wolf, mongoose, and jackal are responsible for rabies transmission in rural areas in that region and may serve as wildlife reservoirs^[^^4^^]^. For rabies prophylaxis, all countries of the region have established the usage of cell culture embryonated egg-based vaccines, although certain member states might have issues for vaccine procurement^[^^5^^]^. Treatment with undesirable nerve tissue vaccines has been discommended by the WHO, and they have almost been discontinued globally. With regard to the rabies control programs, most of the countries need to develop a national strategy plan for rabies control. Certain countries of the region such as Iran, Morocco, Tunisia, and Sudan have already national strategies for rabies control. The main challenge in many of the EMR Member States is that different stakeholders involved in rabies management often work in isolation, and the majority of them do not have a functional reporting system. As a consequence, rabies burden for many countries is limited to estimations from disease modeling^[^^2^^]^. Sometimes, those obtained data  significantly differ from the reported numbers, as could be seen in the case of Egypt, Iran, Iraq, Morocco, Oman, Pakistan, Soudan, Syria, and Yemen^[^^6^^-^^8^^]^. For certain countries, the data are communicated officially to the WHO through regional offices. However, for others, it could only be shared through databases or simply communicated through oral presentations at conferences^[^^6^^,^^9^^]^. It is necessary to have the real image of the disease in the country, and the lack of reliable data concerning rabies may draw rabies to a cycle of neglect and affect the advocacy programs and prioritization of rabies control plans by the authorities. 

**Reiteration of a commitment**

In 2010, the Tripartite (i.e. the WHO, OIE, and FAO), published a concept note on the priority of certain health issues such as rabies, antimicrobial resistance, and avian influenza. Rabies was considered in that document as a good model to be controlled using One Health approach. Due to the nature of the disease, rabies control needs coordinated activities at the human-animal-ecosystem interface^[^^10^^]^. The global meeting organized by the Tripartite in 2015 was a call for such joint action and provided a coordinated vision and approach for global rabies elimination. The conference intended to harmonize actions worldwide and provided adaptable and achievable guidance for countries and regions toward rabies elimination by 2030 (the “zero by 30” goal). Sociocultural, technical, organizational and political activities, as well as resources (STOP-R) were considered as main pillars to reach the defined target. Rabies elimination is a public health good and will contribute to improving the global health; rabies elimination is aligned with the Sustainable Development Goals and the WHO 13^th ^General Program of Work. Rabies burden is considerable (US$ 8.6 bn/year) and ever-increasing, killing one child almost every 20 minutes^[^^2^^]^. The disease elimination is tangible owing to the fact that rabies is fully preventable through proven cost-effective interventions and the availability of all relevant tools. In 2018, the GARC and the Tripartite developed a “Global strategic plan to end human deaths from dog-mediated rabies by 2030” (the GSP), on the basis of comprehensive studies in rabies-endemic countries^[^^11^^]^. The document combined all requirements for rabies elimination and intended to engage 100 countries to develop sustainable rabies elimination plans through three objectives, based on a One Health approach^[^^11^^,^^12^^]^. The core target of the first objective was to mitigate the risk of human rabies through improving awareness and effective vaccination. The other two objectives complemented the collectivity of work through emphasizing the necessity of reliable data, budget sustainability, and multi-stakeholder commitment^[^^11^^]^. Reporting system and systematic vaccination together have shown to be effective in sustained control of rabies burden in a number of countries of the world^[^^13^^]^. Certain countries of the Middle East and Central Asia have succeeded to reduce human rabies cases to or near zero. Those countries have appointed rabies as a notifiable disease for several years, developed bite management centers and put the dog vaccination programs in place. In countries with considerable decrease in human rabies, certain measures such as rate of bite management centers, diagnostic labs, and dog vaccination coverage are remarkable^[^^8^^]^. Since dog bite has been considered as the major cause of human rabies, the MDV has persistently been emphasized on, in the context of rabies elimination. In addition, the efficacy of human rabies elimination through sustained MDV has been demonstrated through elimination programs by the PAHO^[^^14^^]^. It is noteworthy that in such frameworks of action, success largely depends on regional cooperation and political commitment as two pre-requisites, especially for countries that share cross-border activities. Valuable evaluation tools have also been developed such as the SARE, which can remarkably assist countries to know how advanced they are in their efforts^[^^15^^,^^16^^]^. In 2012, the SAARC countries that contribute to 45% of the global burden of human rabies cases launched a Rabies Elimination Project based on a One Health approach, in order to draft a national road map for the member countries until 2020. Member States included Afghanistan, Bangladesh, Bhutan, India, Nepal, the Maldives, Pakistan, and Sri-Lanka. With important variations in their elimination plans, many of those countries emphasized on MDV in their rabies elimination program. Certain SAARC countries have made significant progress and might reach zero human deaths even before 2030, while some others still face significant challenges to reach that goal on time. The progress largely depended on the multipronged attacks to rabies through raising awareness, providing more access to PEP, ameliorating laboratory diagnostic capacity and enhanced MDV^[^^17^^]^. The EMR of the WHO is comprised of 22 Member States from Asia and Africa, facing comparable threats and challenges for rabies control. Several countries of the EMR share common socioeconomic activities and land borders, which could facilitate trespass of rabies reservoir hosts^[^^18^^]^. Similar models of regional cooperation and political commitment could be established for rabies control, based on lessons learned from successful experiments, under the international guidelines. In December 2018, an intercountry meeting was held by the WHO Regional Office in Amman, Jordan, aiming to develop a One Health approach in the region, in order to tackle shared health threats such as zoonotic diseases at the human-animal-environment interface^[^^19^^]^. Countries were instructed to adopt a regional roadmap toward implementation of a One Health approach and tracking of the progress. The essential components of the roadmap were comprised of networking, communication, capacity development, coordinated surveillance, and establishment of a multi-sector collaboration for governance and management^[^^19^^]^. Consistent with the above-mentioned needs for sustained multilateral approaches toward the control of a disease like rabies, acknowledging the available tools by the Member States and putting them into practice would pave the way for the implementation of intervention programs for a zoonotic disease control plan such as global rabies elimination. Nevertheless, according to data from the WHO, certain countries of the EMR would need to take fundamental steps immediately, to reach the “zero by 30” target^[^^6^^,^^9^^]^.

**The global momentum of the WRD**

The WRD is the most important international rabies event, launched in 2007 with the co-sponsorship of the WHO, OIE, and PAHO, and the partnership of GARC and Centre for Disease Control and Prevention (Atlanta, USA)^[^^20^^]^. The WRD tries to encourage groups from the community to the governmental levels to spread the “prevent rabies” message through education, awareness, and action^[21]^. Every year, an annual theme is developed to highlight the significance of the necessary actions for approaching the major milestone that is to eliminate human rabies by 2030^[^^22^^]^. The 14^th^ WRD theme has been named “End Rabies: Collaborate, Vaccinate”. Mass vaccination of dogs has proven to be effective in reducing the burden of human rabies. However, for well-coordinated vaccination campaigns rounds of awareness and education are fundamental and indispensable in any target region. Other essentials to consider, especially in deprived areas that owned dogs are free to roam, would be to encourage responsible dog ownership and to implement waste management. The number of dogs varies among different populations and in varied areas with a dog to human ratio between 10 and 33 dogs for every 100 people^[23]^. Dog population management through birth control methods is another component of rabies management control plans, which should be considered in parallel with MDV, in order to obtain effective results^[^^24^^]^. Rabies control requires orchestrated multipronged practices, comprised of time coordination, advocacy, and planning for vaccination, with education and awareness of the community in the base. Inspired by the constant participation of the Tripartite on this occasion, the WRD motivates the One Health initiatives and creates opportunities for further rabies prevention plans. Many activities with a broad range of magnitude are marked around the world on the occasion of WRD celebration, from an unregistered event to big achievements such as announcement of a rabies-free zone in the Philippines^[^^21^^]^. The Tripartite plus GARC has given a rise to innovative training opportunities and rabies expert consultation meetings in many countries such as Bhutan, Cambodia, China, Iran, Philippines, Thailand, and Sri-Lanka^[^^25^^]^. From one side, those training courses have improved the knowledge and practice on rabies prophylaxis and surveillance and from the other side have supported rabies management sectors to either put into practice or to draft a country-specific control plan. Developing a country-specific plan is challenging and problematic in the context of rabies elimination. In 2012, the SARE tool was developed in order to help countries in the formal evaluation of their current status of rabies control^[^^15^^]^. This user-friendly tool guides countries simply identify gaps as well as strengths and develop a tailored work plan toward freedom from dog-transmitted rabies. Many countries located in the rabies-endemic regions of the world, including certain countries at the WHO-EMR, are at the beginning of the way for the elimination of terrestrial rabies in humans, regardless of the available supporting ground^[^^19^^]^. Countries including Bahrain, UAE, and Qatar in this region have reached an assuring point for rabies control, while for certain other data concerning the rabies, situation are still not readily available^[^^9^^]^. Member States, including Iran, Morocco and Tunisia that have a national strategy for rabies control, are in their way forward^[^^26^^,^^27^^]^. To take any effective action for the implementation of rabies elimination, a functional reporting system to share data is of fundamental importance, and engagement of every stakeholder in this context would be essential. We are in an important period of time when the second phase of the GSP for the fight against rabies is concurrent with the decision of GAVI to add rabies PEP to its 2021-2025 vaccination portfolio^[^^22^^]^. To have more benefit from this stage of the operation against rabies, contribution of knowledge, experiences, ideas, and opinions are necessary. We have to believe what we have learned from our experiences with rabies and define the next steps to make differences at local, national or regional scale. Accordingly, it is essential for any rabies-endemic country to identify and prioritize gaps and find out how to fill them. We should employ the available tools to define priorities, develop clear guidelines for multi-sector collaborations, and draw legal supports in order to reach the target. The WRD provides a unique opportunity and a common platform for advocacy, awareness, and education concerning rabies. It cheers everyone interested in contribution to rabies control to make a difference through thinking globally and acting locally.
